# Recent advances in the development and application of colorectal cancer mouse models

**DOI:** 10.3389/fphar.2025.1553637

**Published:** 2025-05-08

**Authors:** Ting Wang, Zhen Chen, Yuli Zhang, Min Liu, Hua Sui, Qingfeng Tang

**Affiliations:** ^1^ Nanxiang Branch of Ruijin Hospital, Shanghai Jiao Tong University School of Medicine, Shanghai, China; ^2^ Shanghai University of Traditional Chinese Medicine, Shanghai, China; ^3^ The Second Clinical Medical College of Henan University of Chinese Medicine, Zhengzhou, China; ^4^ Jiading Branch of Shanghai General Hospital, Shanghai Jiao Tong University School of Medicine, Shanghai, China

**Keywords:** colorectal cancer, animal model, patient-derived xenografts, carcinogen-induced models, genetically engineered mouse models, metastatic model, spontaneous models, composite animal models

## Abstract

Colorectal cancer (CRC) remains a significant global health challenge, necessitating the development of reliable preclinical models to advance mechanistic understanding and therapeutic innovation. This review comprehensively examines the diverse spectrum of rodent models employed in CRC research, focusing on their unique characteristics, applications, and translational relevance. We systematically evaluate conventional models, including carcinogen-induced models and genetically engineered mouse models (GEMMs), which have been instrumental in elucidating tumorigenic pathways and genetic drivers. Furthermore, we highlight the emergence of patient-derived xenografts (PDX) as a transformative tool for recapitulating tumor heterogeneity and predicting clinical responses. The review also explores metastatic models, which are critical for studying advanced disease, and spontaneous models that mimic natural tumor progression. Additionally, we discuss the growing utility of composite animal models, which integrate multiple methodologies to better reflect the complexity of human CRC. By comparing the strengths and limitations of each model system, this review provides a framework for selecting appropriate models based on specific research objectives. Collectively, these preclinical platforms have significantly advanced our understanding of CRC biology and continue to drive the development of targeted therapies and personalized treatment strategies.

## 1 Introduction

Colorectal cancer (CRC) ranks as the third leading cause of cancer-related mortality globally (Siegel et al., 2024). The pathogenesis of CRC is characterized by a complex interplay of genetic, environmental, and lifestyle determinants that collectively contribute to disease susceptibility (Dekker et al., 2019). Despite significant advancements in CRC research over the past decade, critical challenges remain unresolved, including the early detection of micrometastases and the mechanisms underlying chemotherapy resistance (Shan et al., 2022). In this sense, preclinical animal models have emerged as essential tools for addressing these unresolved scientific questions.

For more than a century, humans have employed rodent models to conduct tumor research. Taking mice as the study model with the benefit of small size, short breeding cycle, high litter size, easy rearing, rapid tumor growth, and rich genetic resources (Carter et al., 2020). In particular, the genetic similarity between mice and humans exceeds 95%, which provides rodent models with a distinctive advantage in the study of CRC (Masopust et al., 2017). Previously, various mouse models have been reported, but each has its own characteristics and application scope. Moreover, there is inter-animal variation in the development of CRC among different mouse models. Therefore, this review provides an overview of the most used CRC murine models, describing their particular benefits and drawbacks.

## 2 Single animal models

**FIGURE 1:**
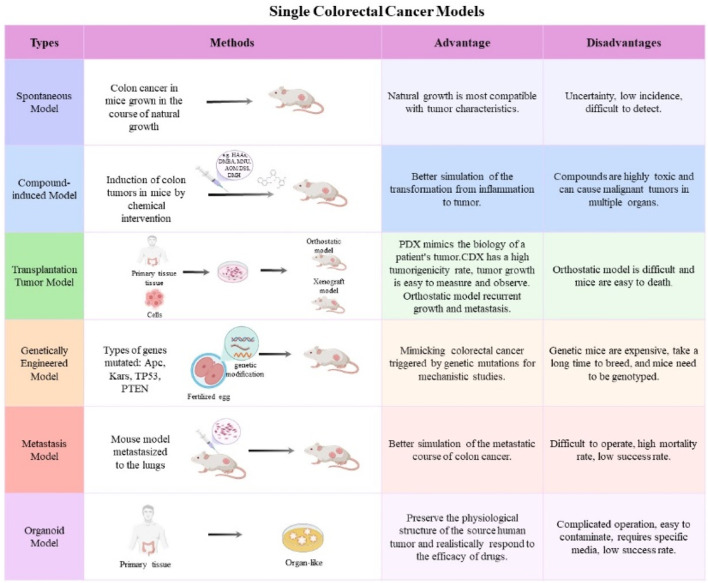
The frequently used single CRC mouse models. The main features and applications of mouse models, including spontaneous models, induced models, transplantable models, genetically modified animals, CRC metastatic tumor models, and organoid models.

The CRC mouse model is an essential instrument for investigating carcinogenesis, development, metastasis, and anti-tumor therapy (Dzhalilova et al., 2023; Pothuraju et al., 2024). Now, single CRC animal models are mostly used in fundamental research, which can be distinctly categorized into spontaneous models, induced models, transplantable models, genetically modified animals, CRC metastatic tumor models, and organoid models (Nakayama et al., 2022; Pothuraju et al., 2024) (Figure 1).

### 2.1 Spontaneous models

At present, there are only a few of instances of spontaneous CRC in rodents or mice. It was found that intestinal hyperplastic lesions, mostly of the type of adenoma or adenocarcinoma, were occasionally found in the intestines of some aged mice (Rowlatt et al., 1969). The spontaneous CRC mouse model can accurately represent the natural occurrence of CRC. Nevertheless, the low incidence rate, the ambiguity of the occurrence time, and the difficulty in detection, which is typically only discovered after endoscopy or animal euthanasia (Nascimento-Gonçalves et al., 2021). This makes it difficult to apply effectively in fundamental research.

### 2.2 Induced models

The mouse model of induced colorectal cancer has a history of more than one hundred years. It continues to be one of the most frequently employed models to this day, and it is currently primarily induced by pharmacological stimulation to produce colonic malignancies in animals (Nascimento-Gonçalves et al., 2021). These models are frequently induced by the following compounds: (1) heterocyclic amines (HAAs), (2) aromatic amines, (3) alkylating agents, and (4) dimethylhydrazine and azoxymethane.

#### 2.2.1 One-step method for inducing CRC

Heterocyclic aromatic amines (HAAs) are highly carcinogenic substances generated during the high-temperature cooking of meat (Barzegar et al., 2019; Le Marchand, 2021). The primary members of this category of carcinogens are 2-amino-3,3-methylimidazo [4,5-f] quinoline (IQ) and 2-amino-1-methyl-6-phenylimidazo [4,5-b] pyridine (PhIP) (Dong et al., 2020). A study demonstrated that 1.5% DSS in combination with PhIP (200 mg/kg) in mice induced colon malignancies with a 100% incidence rate in both male and female mice. This process was closely associated with the P53-dependent DNA damage response in the colon (Yang et al., 2021). Comparable to PhIP, representative compounds in the aromatic amine category, N-methyl-N′-nitro-N-nitrosoguanidine (MNNG) and methyl nitrosourea (MNU), induce colonic mucosal lesions directly via rectal perfusion, resulting in the formation of adenomas and adenocarcinomas. These compounds may serve as optimal local carcinogens due to their lack of requirements for metabolic activation. However, the construction of such CRC animal models requires multiple administrations and is accompanied by strong oncogenic effects that can cause multi-organ malignancies, and is therefore less frequently used (Weisburger et al., 1997; Kawashima et al., 2008; Le Marchand, 2021).

#### 2.2.2 Four-step method for inducing CRC

Comparing to other categories of carcinogens, 1,2-dimethylhydrazine (DMH) and its metabolite azoxymethane (AOM) are the most frequently employed carcinogens for CRC. Lenoir et al. discovered that 60% of the rodents developed colorectal cancer between weeks 20 and 24 after administering DMH continuously for 10 weeks. Furthermore, during the administration period, AOM exhibits greater stability and efficacy than DMH (Lenoir et al., 2016). The method of the combined use of AOM and the inflammatory agent dextran sulfate sodium salt (DSS) has become a classic modeling modality for the study of CRC mechanisms and chemopreventive interventions in CRC research. Briefly, mice are administered an intraperitoneal injection of AOM (10 mg/kg) at the commencement of the experiment. Next, they are required to consume 2.5% DSS for a period of 7 days after a 1-week interval. The subsequent administration of 2.5% DSS for a period of 7 days is performed every 2 weeks. The rodents can be euthanized for subsequent experiments after the completion of three DSS cycles (Angelou et al., 2018; Guo et al., 2021). Although the induction time of AOM-DSS model is longer and the chemical reagents used are more expensive, it mimics the real process of inflammation-induced colorectal tumors *in situ* in the intestines, which is valuable for the study of the mechanism of inflammatory bowel disease-associated colorectal tumorigenesis and progression and related therapeutic strategies. Through the AOM-DSS model, our group found that the traditional Chinese medicine YYFZBJS and natural medicine YTE-17 can prevent colitis-associated tumorigenesis by inhibiting Treg activation (Zhang Y. et al., 2022; Zhang et al., 2024; Sui et al., 2024) and M2 macrophage polarization (Sui et al., 2020a; Chai et al., 2021). The high degree of success rate AOM-DSS model makes it a useful tool in studying human CRC.

### 2.3 Transplantable models

The transplantable colorectal cancer mouse model is a technique that involves the transplantation of tumor cells from cell lines or tumor tissues from patients into mice to induce tumor formation. Two categories can be distinguished based on the location of the transplant: orthotopic transplantation and ectopic transplantation. The two transplantation methods are appropriate for various scenarios and has distinct characteristics.

#### 2.3.1 Ectopic transplantation models

The primary inoculation site for colorectal cancer xenograft models is subcutaneous in mice. This model involves implanting human-derived colorectal cancer cells or tumor tissues from patients into the subcutaneous tissue of immunodeficient mice, leading to the formation of tumor tissue at the inoculation site. Immunodeficient mice are transplanted with tumor cell lines or patient tumor tissues, which are referred to as a cell line-derived xenograft (CDX) and a patient-derived xenograft (PDX), respectively (Georges et al., 2019; Helmer et al., 2021).

The methodology for establishing the CDX mice model is straightforward, with tumor growth occurring over a brief period, typically 1–2 weeks, allowing for the visual observation of subcutaneous nodular protrusions. The incidence of tumor development is about 100%. Furthermore, in the CDX model, the morphology of subcutaneously implanted tumors is relatively uniform, and upon tumor development, the long and short diameters can be measured to compute the tumor’s mass volume, thereby aiding research on the impact of genes and pharmaceuticals on tumor proliferation (Bürtin et al., 2020; Liu et al., 2021a). These characteristics have made CDX mice model the predominant animal model employed in fundamental research on CRC. Our previous studies also demonstrated that colon subcutaneous tumor models are usually used to evaluate therapeutic effects in human cancers (Wei et al., 2023; Wang et al., 2021).

In recent years, the advancement of clinical research has established PDX models as a realistic and trustworthy framework for customized precision medicine, often used to identify appropriate treatment strategies for patients in late disease stages (Perron et al., 2024). The PDX model entails the transplantation of fresh tumor tissue acquired after surgery into immunodeficient mice to precisely replicate the biological traits of the patient’s tumor and the heterogeneity of tumors across various individuals (Xiang et al., 2024). Numerous studies have shown that the CRC PDX model can accurately duplicate the biological features of actual tumors including histology, oncogene expression, and pharmacological response (Cho et al., 2014; Nunes et al., 2015).

#### 2.3.2 Orthotopic transplantation models

The orthotopic transplantation model addresses the limitations of subcutaneous tumor models in accurately representing the orthotopic growth and invasive metastatic traits of colorectal cancers (Rivera et al., 2021). In 1992, Kashtan et al. first introduced tumor cells into the rectal submucosa of mice and observed that after injection, not only did tumors occur locally, but metastasis also occurred in the skin, lymph nodes, and liver tissue (Kashtan et al., 1992). Noteworthy, due to the special location of colorectal cancer, there are two methods of implantation: surgical orthotopic implantation (Surgical orthotopic implantation, SOI) and instillation of cancer cell suspension after destroying the rectal mucosa (Nicolas et al., 2022; Uccello et al., 2023). The establishment and investigation of orthotopic implantation models using cell lines are frequently employed: A subcutaneous tumor is first created by inoculating a cell line subcutaneously, and after the tumor attains a certain size, it is transplanted to the designated spot. The benefit of using cell lines for the creation of orthotopic transplantation models lies in the clarity of their genetic background. The comparability of study findings is enhanced when many researchers use the same cell lines for their investigations. However, the surgical procedure is intricate, and tumors generated using cell lines are unable to form glandular structures. Also, it is simple for rodents to perish and difficult to observe tumor growth (Yang et al., 2022).

### 2.4 Genetically modified mouse models (GEMMs)

GEMMs are the application of contemporary biological genetic engineering techniques to modify the genomes of specific animals (Jackstadt and Sansom, 2016). This modification allows the modified animals to develop or spontaneously form specific diseases and to possess stable hereditary capabilities (Roper and Hung, 2012; Jackstadt and Sansom, 2016). These models may replicate significant gene alterations associated with the initiation of colorectal cancer. With the advancement of bioengineering technology, more and more CRC-related gene alteration sites are being identified (Table 1), with most significant mutations involving tumor suppressor genes such as APC, KRAS, TP53, and PTEN.

**TABLE 1:** Summary of advantages and disadvantages of single mouse models for CRC.

Animal model	Advantages	Disadvantage	References
CDX Model	Easy to establish, short duration, low cost, and convenient for observing tumor size	Tumor microenvironment is not realistic, and tumor cell lines may have contamination or variability	Bürtin et al. (2020), Liu et al. (2021a)
PDX Model	Retains the heterogeneity and genetic characteristics of tumors from different colorectal cancer patients, suitable for precise medicine and selecting anti-tumor drugs	Complex process, expensive process, tumor tissues are difficult to obtain and requires high technical expertise	Perron et al. (2024), Xiang et al. (2024)
Chemically Induced Tumor Model	Mimics the chemical induction process of tumorigenesis, realistic tumor microenvironment, intact immune system, suitable for studies on colorectal cancer pathways and immunotherapy	Long duration, high cost, large individual variability in experiments, and requires high-quality samples; tumor size is difficult to observe	Nascimento-Gonçalves et al. (2021), Le Marchand (2021), Angelou et al. (2018), Guo et al. (2021)
Genetically Modified Mouse Model	High specificity, suitable for research on genes related to colorectal cancer and immunotherapy	Low tumor formation rate, high cost and long duration, and requires sufficient resources (human and material)	Roper and Hung (2012), Jackstadt and Sansom (2016)
Mouse Tumor Cell Transplantation Model	High success rate, short duration, and low cost, suitable for initial studies on colorectal cancer immunotherapy	Tumor cell lines may have contamination or variability	Schroeder, A et al. (2011)
Metastasis Model	Completely or partially simulates the metastasis process of colorectal tumors, useful for studying metastasis-targeted drug strategies	Requires highly skilled techniques and imaging examinations to confirm whether metastasis has occurred	Li et al. (2024b), Rad et al. (2013)

#### 2.4.1 Adenomatous polyposis coli (APC) gene mutation mouse model

The adenomatous polyposis coli (APC) gene, a tumor suppressor gene, directly influences the transition from the G1 to S phase of the cell cycle by participating in the Wnt signaling pathway. The development of intestinal epithelial cells is dysregulated because of APC mutations, which ultimately leads to colorectal cancer (Ren et al., 2019). In 1990, Moser and colleagues employed N-ethyl-N-nitrosourea (Enu) to induce a point mutation in the Apc gene of rodents at the 850th amino acid. This mutation resulted in the conversion of TTG to the stop codon TAG, thereby removing the tumor-suppressing function of the gene. By the age of 15 weeks, these Apc^Min/+^ rodents had developed numerous adenomas throughout the entire intestine (Moser et al., 1990). It is evident that the mutated gene demonstrates complete penetrance and dominant expression.

For 30 years, Apc^Min/+^ rodents have been extensively employed in research on tumor therapy, gene function testing of intestinal malignancy, and chemical prevention. In our studies, we also detected higher levels of PCNA and Ki67 in the intestinal tumor, suggesting that the alterations in the intestinal microenvironment are an important reason in malignant tumor carcinogenesis (Sui et al., 2020b; Sui et al., 2024). Additionally, to replicate the malignancy of clinical patients in Apc^Min/+^ mouse polyps, researchers have developed many other models with truncated Apc alleles. One such model is the Apc1322T mouse model, which is strikingly like the mutation that occurs at codon 1,309 of the human cancer Apc gene. The Apc protein is entirely absent in this model, and the condition is substantially more severe than that of Apc^Min/+^ rodent polyps. This is evidenced by the condition’s earlier onset, larger and more numerous polyps, and the development of poorly differentiated adenomas. Meanwhile, the malignancies demonstrate a greater degree of Paneth cell differentiation and a higher incidence of crypt fission (Pollard et al., 2009; Mieszczanek et al., 2019). Furthermore, there is the Apc^Δ14/+^ mouse model, which directly deletes exon 14. This model demonstrates a more severe tumor phenotype, which is characterized by a higher burden of colon tumors, increased lethality, and muscle invasion (Colnot et al., 2004; Bonnans et al., 2012).

#### 2.4.2 KARS gene mutation murine models

Kras is one the most often mutated oncogenes in malignant tumors, with a mutation incidence of roughly 40% in colorectal cancer patients. Upon the occurrence of a Kras mutation, GTP hydrolysis is impaired and/or nucleotide exchange is augmented, resulting in the accumulation of active Kras, which facilitates the persistent activation of downstream signaling pathways, thus increasing tumor cell proliferation (Li et al., 2012; Zhu et al., 2021). Researchers interbred Kras gene mutant mice with Apc gene mutant mice to expedite the development of intestinal cancer in the subjects (Maitra et al., 2019). The AhCre Apc^fl/+^ Kras^+/LSLV12^ mice showed that the activation of Kras (V12) after Apc deletion results in a heightened incidence of tumors (Sansom et al., 2006). The subsequent investigation revealed that the tumor diversity in Kras^G12V^Apc^1638N/+^ mice increased tenfold, resulting in less apoptosis, accelerated progression and quicker tumor proliferation (Bürtin et al., 2020). This contributed to significantly higher morbidity and mortality rates.

#### 2.4.3 TP53 gene mutation mouse model

TP53 is a critical tumor suppressor gene, and its mutations are relatively prevalent in CRC, with an incidence of approximately 34% (proximal colon tumors) and 45% (distal colon tumors). The Apc^Min/+^p53^−/−^ mice model, which combines Apc gene mutations with homozygous p53 deletion, has been shown in early research to marginally increase the prevalence of gastrointestinal cancers (Sakai et al., 2018). In 2017, Nakayama et al. demonstrated that an additional nuclear accumulation-type p53 mutant (p53R270H) can promote malignant progression of intestinal tumors by generating complex tumor gland structures and gaining invasiveness, alongside a notable increase in the quantity of myofibroblasts in the stroma (Nakayama et al., 2017).

#### 2.4.4 PTEN gene mutation mouse model

In general, the normal structure of the intestinal epithelium and the body’s homeostasis are insignificantly affected by the specific loss of PTEN in adult or embryonic epithelial cell populations. Nevertheless, the rapid development of adenocarcinoma can be facilitated by the loss of PTEN, which can accelerate tumorigenesis by increasing Akt activation, because of the Apc gene deletion (Marsh et al., 2008). Additionally, the activation of Kras, in conjunction with the loss of PTEN, disrupts intestinal epithelial homeostasis in VillinCreERT Apc^fl/+^Pten^fl/fl^Kras^LSL/+^ mice, resulting in the formation of hyperplastic polyps and the further promotion of dysplastic sessile serrated adenomas and serrated-featured metastatic adenocarcinomas (Davies et al., 2014).

Compared to chemically induced and tumor cell transplantation models, genetically engineered animals can better mimic the dynamic interactions between tumor cells, stroma, and the immune system, as well as the response to treatment, and are more suitable for gene function studies. Genetically engineered animal models are more appropriate for the study of gene functions because they can more accurately simulate the dynamic interactions between tumor cells, stroma, and the immune system. However, the tumors in these models predominantly manifest as polypoid adenomas, and they are primarily distributed in the small intestine rather than the colon, which is distinct from the majority of clinical patients with Apc mutations.

Additionally, mouse models that are created by incorporating additional gene mutations based on Braf mutations have the potential to develop more aggressive serrated colorectal cancer. However, the time taken for tumor formation is longer. Consequently, emerging evidence suggests that prolonged latency periods in rodent models may facilitate the accumulation of somatic mutations, potentially resulting in tumor progression patterns that more closely recapitulate the molecular and phenotypic characteristics of human colorectal cancer (CRC). This extended tumorigenesis process could establish more clinically relevant animal models for CRC research, particularly in terms of genetic heterogeneity and tumor evolution.

### 2.5 Colorectal cancer metastasis mouse models

Metastasis is a main cause of death for patients with CRC, hence, utilizing mouse models to recapitulate the clinical characteristics is crucial for studying the underlying mechanism and for developing effective treatment against metastasis. Although substantial CRC mouse models have been established, models that can develop features of metastasis remain rare.

#### 2.5.1 Cell and organoid xenotransplantation

Subcutaneous injection of cancer cells is commonly used in xenograft models due to its convenience and high success rate to induce tumor formation, yet it fails to produce metastasis, while orthotopic injection of CRC cells into specific organs such as caecum, tail vein, and spleen can lead to metastasis in liver, lung, and bones (Li et al., 2022). As used in our previous study, six-week-old nude mice injected the CRC cell line HCT116 with ectopic ITGBL1 expression into nude mice and reported a 100% incidence rate of lung metastasis in these xenograft mice (Ji et al., 2020), indicating that MiR-200c/MDR1 can promote the invasion and migration of CRC cells. Notably, a major problem of xenograft models is the incomplete tumor microenvironment, of which the tumor barrier such as the basement membrane is lacking in xenograft mice.

#### 2.5.2 Transgenic mouse model of metastasis

In addition to transplantation mouse models, metastasis can also be induced in transgenic mice, although the latency is long with low penetrance. It was reported that heterozygous deletion of Braf ^LSL–V637E/+^ leads to full progression from serrated hyperplasia, adenoma, and finally to metastatic carcinoma (Rad et al., 2013). However, the latency of this transgenic model is long, and the metastasis rate is only 20%. Altogether, unless major breakthroughs are achieved, transgenic mouse models remain a minor alternative of xenotransplantation in the study of metastasis (Table 2).

**TABLE 2:** Genetically engineered mouse models of colorectal cancer.

Animal model	Tumorigenesis period	Tumor characteristics	References
Apc^Min/+^	15 Weeks	The condition is primarily characterized by multiple adenomas in the small intestine, which can also occur in the colon and rectum. These are mostly tubular villous adenomas or tubular adenomas, with a low degree of malignancy	Ren et al. (2019)
Apc^1638N^	20 Weeks	Tumors are few (<10), the latency period is long, and they develop into adenocarcinomas as they infiltrate into the submucosa	Ecker et al. (2021)
Apc^Min/+^p53^−/−^	Within 90 Days	Dense connective tissue, local invasion, and the tumor infiltrates into the underlying muscularis mucosae	Liebl and Hofmann. (2021)
AhCre Apc^fl/+^Pten^fl/fl^	7 Days after induction	Most are invasive adenocarcinomas, caused by severely dysplastic adenomas, with significant malignant invasion of the submucosal layer	Howe et al. (2018)
Apc^fl/fl^ Pik3ca^p110^ Apc^fl/fl^ Kras^G12D/+^ Pik3ca^p110^	3 Weeks after Cre induction	80% of the tumors are invasive adenocarcinomas, many of which extend beyond the muscularis propria, involving the serosa, with a small number of retroperitoneal aortocaval lymph nodes and liver metastases	Hadac N et al. (2015)
Apc^Min/+^ Smad3^−/−^	60 Days	The tumor invades through the submucosa and into the local muscle tissue	Sodir et al. (2006)
Apc^+/1638N^ Smad4^+/E6sad^	Transmodel as early as 3 Months; Cis model at the earliest 3 Weeks	Tumors in the trans model are primarily villous or tubulovillous adenomas, with a higher degree of dysplasia and malignancy, mainly distributed in the duodenum; the cis model is characterized by significant anemia and splenomegaly, with a greater number of tumors and shorter survival periods	Takaku et al. (1998)
LSL-Kras^G12D^ Tgfbr2^IEKO^	Within 22 Weeks	Most are adenocarcinomas, about 15% of mice develop obvious metastatic lesions in regional lymph nodes or lungs	Trobridge et al. (2009)
VillinCre Braf^LSL−V637E/+^	10 Months	Serrated adenoma, including crypt elongation and serrated eosinophilic cell adenoma epithelium	Rad et al. (2013)
Vil-Cre Braf^LSL−V637E/+^ p16^Ink4a^	10–20 Months	Serrated tumors, 12% of mice have developed metastatic tumors	Rad et al. (2013)
Vil-Cre; Braf^V637E/+^ p53^LSL−R172H/+^	10–20 Months	Serrated tumors, 25% of mice have developed cancer that has metastasized to local lymph nodes, pancreas, or lungs	Rad et al. (2013)
Apc^Δ716^Kras^G12D^	13–16 Weeks	Activated Kras increases tumor diversity, with a significant increase in the number of polyps, and no invasive tumors were found	Sakai et al. (2018)
Apc^Δ716^ Kras^G12D^ Fbxw7^−/−^	13–16 Weeks	Epithelial-mesenchymal transition occurs, accelerating tumor growth	Sakai et al. (2018)
Apc^Δ716^ Kras^G12D^ Tgfbr2^−/−^	13–16 Weeks	The number of myofibroblasts expressing αSMA increases, and the incidence and diversity of lymphatic vessel invasion and liver metastasis are significantly higher	Sakai et al. (2018)

### 2.6 Organoid models

Organoids are three-dimensional *in vitro* cultures derived from the self-organization of organ precursor cells via stem cell technology (Yang et al., 2023). Organoid technology is highly comparable to *in vivo* tissues in terms of tissue structure due to its ability to replicate the gene expression profile of the source tissue and the core characteristics and functions of the corresponding organ (He et al., 2020). Patient-derived tumor organoids (PDTOs) are a novel instrument for the study of tumors, providing substantial benefits in the investigation of the mechanisms of tumorigenesis and development (Thorel et al., 2024). It employs stem cells to generate three-dimensional (3D) tissue-like structures *in vitro* via self-organization, self-renewal, and differentiation, thereby preserving the physiological architecture and functional attributes of the originating tumor, while retaining the distinctions among tumors from various patients (Moss et al., 2024).

The PDTO model is effective in assessing the *in vivo* efficacy, toxicity, and adverse reactions of drugs, which enhances the efficiency of drug development and the success rate of clinical trials (Thorel et al., 2024). This approach has a wide range of potential applications in personalized medicine, new drug development, and tumor drug screening. In mouse models and organoids derived from cancer cells of human colorectal cancer patients, Gou et al. confirmed that the traditional Chinese medicine Pianzihuang can enhance intestinal barrier function, inhibit carcinogenic and pro-inflammatory pathways, improve the gut microbiota and its metabolites, and suppress the occurrence of colorectal cancer (Gou et al., 2023). Utilizing the PDTO sample library, a study in 2022 conducted a high-throughput screening of more than 500 bispecific antibodies, which enabled the identification of MCLA-158, a bispecific antibody (Herpers et al., 2022). This antibody is capable of specifically degrading the EGFR protein in G protein-coupled receptor 5-positive (LGR5^+^) tumor stem cells, thereby effectively inhibiting the formation of colorectal cancer organoids and preventing metastasis. Additionally, the functionality of typical stem cells is not disrupted by this antibody. This investigation established the theoretical framework for the utilization of organoids in the development of pharmaceuticals.

With the help of organoid modeling, our group also demonstrated that AhR significantly enhances stemness and migration of intestinal cancer cells by promoting the activation of the Wnt/β-catenin signaling pathway in intestinal cancer cells (Zhang et al., 2023). However, another obvious limitation of PDTO is the lack of vascular system and tumor microenvironment. Nevertheless, the absence of a fully developed vascular network structure in tumor organoids can restrict the provision of oxygen and nutrients to the cells within the organoids, resulting in apoptosis and a decrease in vitality.

PDTOs are more physiologically and pathologically relevant than traditional models, even though there are still some challenges at this stage. In addition to preserving the tumor heterogeneity of the patients, they can more effectively summarize the molecular, cellular, and tissue pathological phenotypes of the source tumors. There is significant potential for PDTO testing to assist in the development of personalized treatment.

## 3 Composite animal models

**FIGURE 2:**
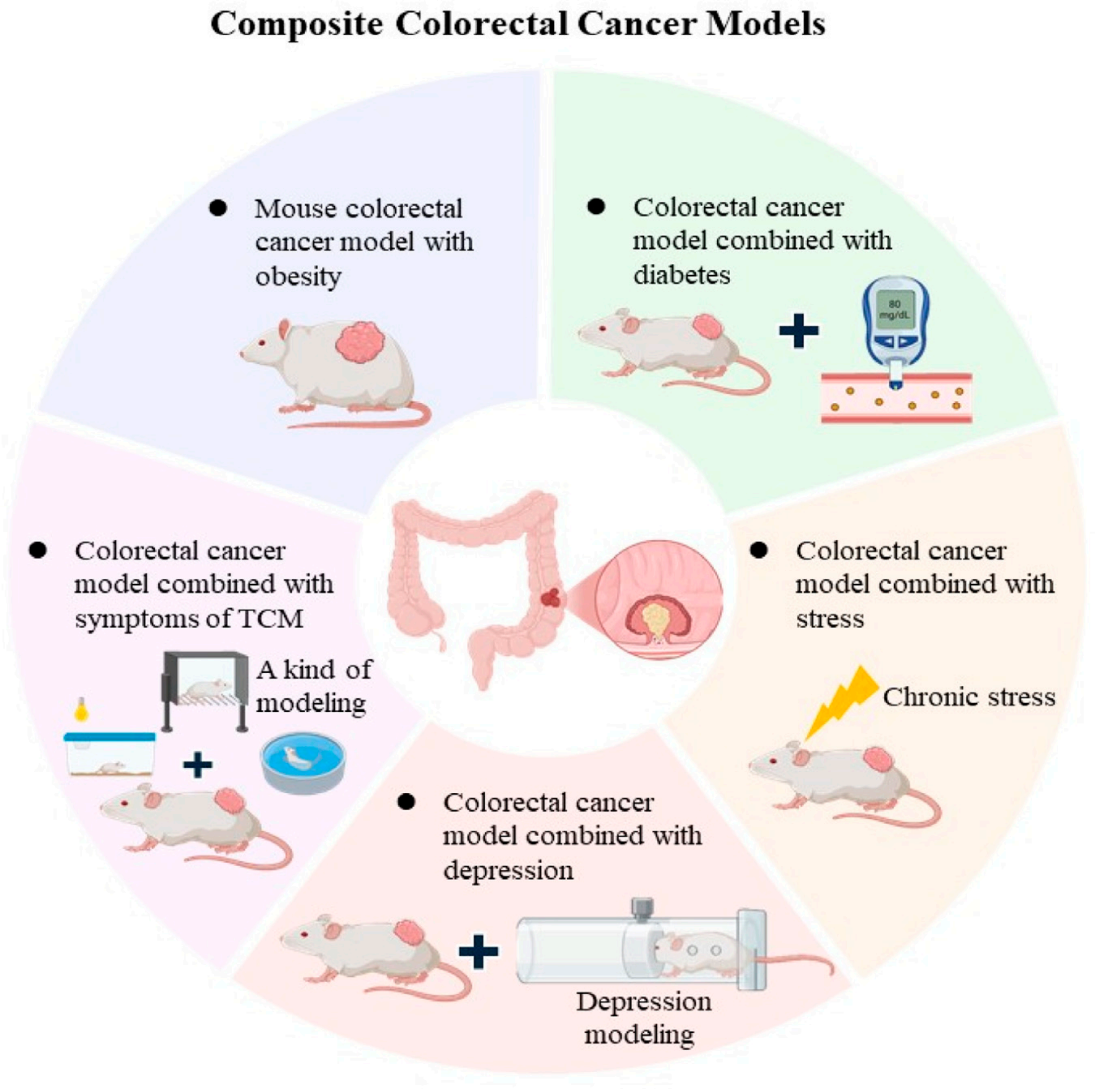
Composite CRC Models The main features composite CRC models, including obesity-associated models, diabetes (Metabolic Syndrome) combined with CRC models, stress and depression combined with CRC models.

In biomedical research, two different types of animal models are employed in an integrated approach, that in order to more fully simulate human diseases or physiological processes (Anisimov, 2016). CRC is a disease caused by multiple genes, with the in-depth study of colorectal cancer, some new composite animal models have emerged, including gene double knockout model, induced colorectal cancer model combined with obesity model, diabetes model, etc (Barker et al., 2018; Neumann et al., 2021; Lv et al., 2023). In addition, with the changing living environment in modern society, stress, depression, and exercise also have an important impact on colorectal cancer, and the study of the corresponding composite model is becoming more and more popular (Shao et al., 2021; Cheng et al., 2022; Lv et al., 2023). Therefore, the role and influence of these factors should be considered in the prevention and treatment of colorectal cancer comprehensively to develop personalized treatment plans and interventions (Figure 2).

### 3.1 Obesity-associated CRC models

Obesity is a chronic metabolic disease, which has experienced a significant rise in global prevalence over the last 50 years (Zhou et al., 2024). Epidemiological studies demonstrate that chronic low-grade inflammation resulting from obesity markedly increases the risk of CRC (Gonzalez-Gutierrez et al., 2024). Therefore, the establishment of a composite animal model of obesity and colorectal cancer can help to reveal the mechanisms of colorectal cancer development and progression in the case of obesity interventions, including the roles of obesity-induced intestinal microecological imbalance, inflammatory response, and insulin resistance in colorectal cancer (Chen et al., 2021; Chen et al., 2024).

#### 3.1.1 Obesity-induced tumorigenesis and metastasis

At present, the existing composite models of obesity-associated CRC are mainly developed through the introduction of chemical carcinogens or the transplantation of tumor tissues into obese animal subjects. Additionally, genetically obese mouse models, such as ob/ob or db/db mice, have been utilized in combination with CRC models to investigate the role of obesity-related genes, particularly those involved in the leptin signaling pathway, in tumorigenesis. Researchers have induced CRC in obese mice through the administration of AOM. It induced CRC in obese mice through the administration of AOM. The findings indicated that obese mice with elevated leptin (LEP) expression showed heightened vulnerability to AOM-induced CRC (Singh et al., 2020).

#### 3.1.2 Mechanisms of obesity-induced tumorigenesis and metastasis

##### 3.1.2.1 Obesity-induced the inflammatory signaling

**FIGURE 3:**
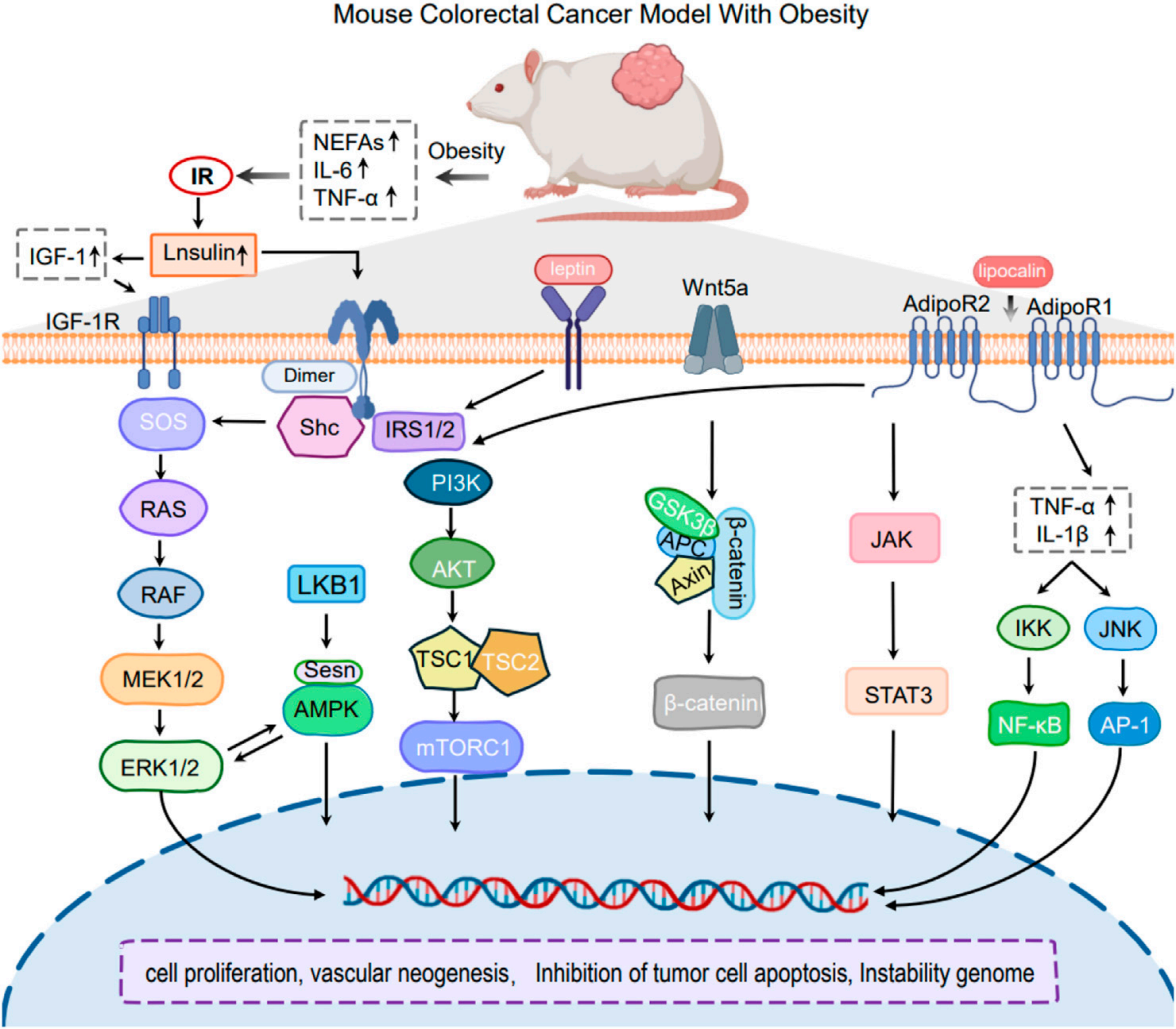
Mechanism of Obesity-induced CRC mouse model.

The accumulation of white adipose tissue is recognized as a significant pathophysiological change in the progression of obesity (Reyes-Farias et al., 2021). Accumulated adipose tissue activates inflammatory signals such as endoplasmic reticulum stress, oxidative stress, and NLRP3 inflammasome, which in turn releases multiple cytokines that act in the tumor microenvironment (TME) and promote tumorigenesis and further progression (Ahechu et al., 2018; Bouche and Quail, 2023) (Figure 3).

###### 3.1.2.1.1 Cytokine Release

Cytokines, a type of tiny soluble protein that mediates communication between immunological and somatic cells, play an important role in obesity-related CRC. It has been found that in obese individuals, the expression of tumor necrosis factor-alpha (TNF-α) is markedly increased in white adipose tissue, resulting in heightened local concentrations. Obesity-induced enhancement of TNF-α expression facilitates the development of colorectal cancer through various mechanisms. TNF-α has been shown to causally mediate Wnt signaling associated with obesity, indicating that inflammation-driven Wnt signaling may be a mechanism underlying colorectal carcinogenesis linked to obesity (Liu et al., 2012; Guo et al., 2020). Likewise, obesity is associated with altered expression and functions of additional cytokines, including interleukin-6 (IL-6), interleukin-1 (IL-1). Cytokines are involved in the activation of systemic chronic inflammation via pathways such as NF-κB/STAT3 and PI3K/AKT, which contribute to the initiation and progression of colorectal cancer CRC (Jin et al., 2021; Scheurlen et al., 2022).

###### 3.1.2.1.2 Regulation of Local Immune Cells

A large amount of data shows that the expansion of white adipose tissue increases the activation and recruitment of immune cell subpopulations, thereby promoting the development and progression of CRC. Various immune cells located at the intestinal epithelium play pivotal roles in maintaining gut homeostasis. In adipose tissue, infiltrating immune cells, such as macrophages, B cells, T cells, are the second largest population after adipocytes and play critical roles in regulating tissue function and homeostasis (Man et al., 2022). The past decade has revealed several key regulators of macrophage polarization, including the signal transducer and activator of transcription family, the peroxisome proliferator-activated receptor gamma, the CCAAT-enhancer-binding proteins (C/EBP) family, and the interferon regulatory factors (Li et al., 2018; Fortelny et al., 2024). Notably, the polarization of macrophages is affected under obesity stress, and T cell and adipocyte activity, signaling of regulatory proteins and changes in DNA methylation status contribute to those alternations. In addition, pro-inflammatory factors modulate immune cells in the TME, inducing immune dysfunction in cells such as neutrophils, eosinophils, mast cells, etc. (Russo et al., 2021; Izquierdo et al., 2022; Scheurlen et al., 2022), which ultimately promotes the development and progression of CRC.

##### 3.1.2.2 Inflammation-related pathways

###### 3.1.2.2.1 Hypoxia-associated inflammation

In the state of obesity, the expansion of adipose tissue disrupts the balance of oxygen supply and demand in adipocytes, particularly in those that are situated far from the vasculature (Engin, 2024). Hypoxia promotes the release of pro-inflammatory mediators from adipocytes and inhibits the synthesis of the anti-inflammatory adipokine adiponectin (ADPN) (Trayhurn et al., 2008). This leads to the infiltration of macrophages and the apoptosis of adipocytes. The progression of obesity-related complications is significantly influenced by hypoxia. Hypoxia plays a critical role in CRC by inducing angiogenesis, activating proliferative signaling pathways, and modifying tumor cell metabolism, thereby altering the TME.

###### 3.1.2.2.2 NLRP3 Inflammasome

It demonstrated that the NLRP3 inflammasome, which is responsible for the initiation and progression of CRC in rodents, is abnormally expressed in response to a high-fat diet (Zhang et al., 2024). Nevertheless, the significance of NLRP3 in CRC has yielded conflicting results. NLRP3 or caspase-1 mutant rodents are reported to have reduced levels of the effector molecules IL-1β and IL-18, which results in a decrease in the incidence and progression of CRC. However, NLRP3 plays a multifunctional role in CRC through its downstream mediators IL-1β and IL-18, with evidence indicating that these effects are tissue-specific. Despite this, the pro-colorectal cancer function of NLRP3 is now corroborated and validated by most investigations.

###### 3.1.2.2.3 Other inflammatory pathways

Other inflammation-related pathways implicated in CRC include the NF-κB pathway, Toll-like receptor (TLR) signaling pathway, and Janus kinase (JAK) pathway (Sikalidis and Varamini, 2011). These pathways provide a persistent inflammatory environment that facilitates carcinogenesis and progression in obesity-related colorectal cancer.

##### 3.1.2.3 Obesity-induced hormone release

Adipose tissue is gradually acknowledged as a significant endocrine organ, capable of emitting a diverse array of hormones and signaling molecules (Kawai et al., 2021). A variety of obesity-related hormones, including leptin, adiponectin, insulin/insulin-like growth factor (IGF) family members, and sex hormones, have been identified in recent studies (Nikolaou et al., 2019; Nguyen and Shanmugan, 2024), as contributing to the colon carcinogenesis and poor prognosis of CRC. These hormones promote the progression of CRC by activating pathways such as JAK-STAT3, Ras-Raf-MEK-ERK, and PI3K/AKT through their interactions with their receptors (Massaro et al., 2020; Bahar et al., 2023; Olszańska et al., 2023).

###### 3.1.2.3.1 Leptin (LEP)

LEP is synthesized by adipocytes under normal circumstances, and its levels in the bloodstream are directly proportional to fat stores (Friedman and Halaas, 1998). Obesity is usually associated with excessive leptin release, and chronic obesity can be caused by high levels of leptin that continue to stimulate leptin receptors (LRs), resulting in resistance to leptin (Izquierdo et al., 2019). Notably, elevated circulating levels of leptin were found to significantly increase the risk of colon carcinogenesis in obese patients (Ye et al., 2020). Obese mice expressing high levels of LEP showed susceptibility to AOM-induced colon cancer, while colon cancer rats were intervened with LEP, which suppressed colon cancer precancerous lesions (Singh et al., 2020). Leptin activates signaling pathways in the cellular network, including PI3K/JAK signal transducer and activator of transcription (STAT) pathways through binding to the leptin receptor, through inflammation, cell proliferation, inhibition of apoptosis and angiogenesis, etc (Obradovic et al., 2021).

###### 3.1.2.3.2 Adiponectin (ADPN)

ADPN is a primary hormone produced by adipose tissue that has the potential to enhance insulin sensitivity and energy expenditure. Under high-fat diet conditions, Fenton JI and colleagues investigated whether ADPN inhibits the development of colorectal cancer. They discovered that mice lacking adiponectin and adiponectin receptors 1/2 exhibited a substantially higher incidence of hyperlipidemia and colonic lesions than control mice after being fed a high-fat diet (Fenton et al., 2008). Further study confirmed that Polyp formation may be inhibited by exogenous adiponectin supplementation (Otani et al., 2010). In addition, most tumor cells express the lipocalin receptors AdipoR1 and AdipoR2, and binding of lipocalins to their receptors leads to activation of intracellular pathways that negatively regulate proliferation, such as the AMP-activated protein kinase (AMPK) signaling network, and inhibition of those pathways that stimulate cell division and growth, including PI3K/AKT/mTOR, JAK/STAT3 and extracellular regulated protein kinases 1/2 (ERK1/2) (Kim et al., 2010; Kim et al., 2024).

###### 3.1.2.3.3 Sex Hormones

Sex hormones are steroid hormones produced by the gonads (e.g., testes, ovaries) in animals, as well as by tissues such as the placenta and the reticular zone of the adrenal cortex, and they are chemically classified as lipids (Nilsson et al., 2001; Hamilton et al., 2017). In the condition of obesity, estrogens have a more significant influence on colon cancer than androgens. In an inflammatory state, estrogen promotes colon cancer, and further studies have shown that estrogen also promotes tumor growth in already tumor-bearing mice, which may be due to estrogen exacerbating inflammation through IL-6 (Ye et al., 2020). However, the protective role of estrogen against colon cancer is currently controversial. It found that estrogen reduced the risk of colorectal cancer by about 40% (Lin and Giovannucci, 2010).

### 3.2 Diabetes (metabolic syndrome) combined with CRC model

Diabetes, a prevalent chronic metabolic disorder, may inflict significant harm on the body owing to sustained hyperglycemia, which includes an increased risk of developing CRC (Yu et al., 2022; Lawler et al., 2023). Research indicates that the incidence of CRC in diabetic individuals exceeds that of the general population by almost 1.5 times, with type 2 diabetes mellitus (T2DM) identified as an independent risk factor for CRC (Guraya, 2015; Lawler et al., 2023). Most recent investigations use models in which T2DM and cancer are concurrently produced in mice (Cao et al., 2013; Zhou et al., 2018). Nevertheless, it is uncertain whether diabetes accelerates tumor progression or tumors exacerbate diabetes due to the varying onset timelines of the two conditions.

#### 3.2.1 Effects and mechanisms of diabetes on CRC

To verify the effect of diabetes on CRC, most studies have employed animal models with specific compound factor stimulation or double gene knockouts animal. For instance, to study the effect of colorectal cancer growth in the high NE state of type 2 diabetes mellitus, a composite model of type 2 diabetic colon cancer mice was established by feeding with high-fat chow and intraperitoneal injection of 1% streptozotocin (STZ). The results demonstrated that, compared to normal CRC-bearing mice, T2DM mice exhibited faster tumor growth, larger tumor size, heavier tumor weight, and shorter survival times (Chen, 2017). These findings indicate accelerated tumor progression under diabetic conditions.

#### 3.2.2 Effects and mechanisms of CRC on diabetes

Cancer itself may lead to metabolic abnormalities, including disturbances in glucose metabolism, which may increase the risk of diabetes mellitus. Some researchers established a Type 2 Diabetes Mellitus model for colorectal cancer, and revealed that the activation of ERK1/2 and JNK signaling by insulin and IGF-1, at least in part, is responsible for the development of colon cancer with T2DM (Teng et al., 2016). For example, Dipeptidyl peptidase-4 (DPP-4) inhibitors are a recently developed class of antidiabetic pharmaceuticals that are garnering attention for their additional benefits, including enhanced pancreatic function, a reduced risk of hypoglycemia, and weight reduction. Yorifuji et al. have shown that sitagliptin, a clinically used DPP-4 inhibitor, suppresses the occurrence of CRC in T2DM mice through the GLP-1 pathway in animal studies using mice (Yorifuji et al., 2016). These results indicate that DPP-4 inhibitors may have a dual function in the prevention of CRC and the management of diabetes.

### 3.3 Stress combined with CRC model

When the body’s internal environment is disrupted or when it is confronted with a threat to homeostasis, stress is an adaptive response that is produced. Stress can be classified as acute or chronic stress, depending on the duration of the associated stressor (Chu et al., 2024). Different stress responses involving the sympathetic nervous system and adrenal medullary hormone systems are induced by the nature and intensity of the stressor, as well as individual life experiences, from a physiological perspective. Numerous studies have shown that stress is a substantial risk factor for the promotion of tumors (Guan et al., 2023; Chu et al., 2024).

Recent research has demonstrated that chronic stress can contribute to the progression of cancer by promoting inflammation. A protracted stress-induced increase in leukocyte infiltration in the intestines was observed in an animal model of colorectal cancer. The expression and secretion of inflammatory cytokines, including interferon-γ (IFN-γ), interleukin-18 (IL-18), IL-2, and IL-12, were elevated in rats that were subjected to long-term, unpredictable mild stress (Jiang et al., 2024). As a result, the risk of intestinal microbiota dysbiosis was elevated, and intestinal permeability was increased. These results indicate that chronic stress may contribute to the development of colorectal cancer by exacerbating inflammation and disrupting intestinal homeostasis.

### 3.4 Depression combined with CRC model

Depressive disorder (Depressive disorder) is a common mental disorder, mainly characterized by depressed mood, slowed thinking, psychomotor inhibition, difficulty concentrating, and easy mental and physical fatigue. It has a substantial effect on the social functioning of patients and is one of the psychological disorders that is most strongly associated with suicidal ideation and a high risk of suicide. Many clinical observations demonstrated that cancer patients, especially those with malignancies such as CRC, are more prone to develop depression (Li C. et al., 2024). The severity of the disease, treatment outcomes, and functional impairments are frequently associated with the degree of depression in CRC patients (Howren et al., 2022). The interaction between cancer and depression frequently results in a pernicious cycle, which exacerbates both physical and psychological health.

The currently employed model of depression combined with colon cancer is basically the administration of depressive stimuli on a variety of colon cancer models, with successful model building demonstrated through behavioral phenomena (Rondepierre et al., 2024). In line with this, we combined depression in a mouse model of colorectal cancer and found that depressed mice were more likely to develop colon cancer liver metastases compared to normal colon cancer mice (Shao et al., 2021).

### 3.5 Integration of traditional chinese medicine syndromes with CRC models

In recent years, with the development of the theory of disease-evidence combination animal models, some researchers have constructed animal models of colorectal tumors characterized by the integration of disease and syndrome under the guidance of TCM theory. These models can be categorized into six categories based on TCM syndromes: cold-heat syndrome, phlegm syndrome, liver depression syndrome, spleen deficiency syndrome, blood stasis syndrome, and dampness -heat syndrome (Table 3). Currently, the majority of CRC models based on TCM theory are designed as disease-syndrome overlapping models. These models integrate CRC animal models with TCM etiological and pathophysiological methodologies. However, these models exhibit certain limitations: they essentially amalgamate the concepts of “disease” and “syndrome” without establishing an intrinsic link between the emergence of the “syndrome” and the disease progression. This raises significant challenges in ascertaining whether the “syndrome-based modeling approach” plays a direct or indirect role in the tumorigenesis of CRC. Moreover, replicating the intricate correlation between “syndrome” and “disease” as observed in clinical settings remains a formidable task.

**TABLE 3:** Different syndromes of traditional Chinese medicine animal model.

Major syndrome	With patterns	Model Animal	Modeling method	Modeling period	Phenotype (symptom characteristics)	Characteristic pathological alterations	Underlying molecular changes or mechanisms	Representative prescription	References
Heat-Cold Syndrome		Wistar Rat	Heat pattern:DMH s.c.with Capsaicin and ethanol Mixture i.g.; 24–38 Weeks	24–38 Weeks	①	Irregular glandular architecture, intramucosal carcinoma of colorectum		Zuojinwan,Retro-ZuojinWan	Nong, 2022; Qin (2022), Chen et al. (2023)
			Cold Pattern:DMH s.c. and i.g. 0°C Ice Water, Soaking in 10°C Water; 24–38 Weeks	24–38 Weeks	②	Glandular architecture disorganization, colorectal mucosal invasive carcinoma	The serum SDH, LDH, Ca^2+^- Mg^2+^- ATP, and Na + - K + - ATPase activities in the cold syndrome model group were significantly reduced, while the protein expression of PD-1, PDL1, TIM-3, and HMGB1 in colon tissue was upregulated, and the expression of CD4 and CD8 proteins was downregulated		Nong, 2022; Qin (2022), Chen et al. (2023), Sui et al. (2014)
Phlegm-Dampness Syndrome		C57BL/6J Mice	AOM i.p. and DSS Free Drinking with High-fat Diet; 26 Weeks	26 Weeks	③	Cellular atypia in mucosal and submucosal layers, goblet cell architectural disarray	M2 polarization of macrophages	Erchen decoction	Zhang, 2020; Liu (2021)
		C57BL/6J Mice	MC-38 Cell s.c. and High-fat Diet; 8 Weeks	8 Weeks	③	Increased adenomatous polyps	Decreased proportion of CD3^+^, PD1^int^, TIM3-, CD8+T cells	Dahuang Fuzi Baijiang Powder	Xu (2022)
		APC^min/+^Mice	Genetically Engineered Mice and High-fat Diet; 16 Weeks	16 Weeks	③	Increased adenomatous polyps		Dahuang Fuzi Baijiang Powder	Xu (2022)
		BALB/CNude Mice	HCT-8 Cell s.c. and High-fat Diet, Colds and Dampness Stimulation; 2 Weeks	2 Weeks	②③	Increase in the degree of tumor malignancy	Elevated levels of TC, LDL-C, and HDL-C in serum		Wu (2019)
Stagnation Syndrome		Wistar Mice	DMH s.c. with Restraint Immobilization Method; 20 Weeks	20 Weeks	④		Decreased levels of neurotransmitters NE, E, and 5-HT in plasma	Sini Powder	Zhao (2001)
		BALB/CNude Mice	Spleen Injection of HT-29 Cells to Induce Liver Metastasis Model with Restraint Immobilization Method; 5 Weeks	5 Weeks			Elevated levels of NE, E in serum and NE in spleen Elevated protein expression of TGF - β, IL-6, VEGF, and MMP-9	Xiao Yao San	Zhao, 2016; Ma et al. (2017)
	with spleen deficiency syndrome	SD Rat	DMH s.c. with Anger Stress Method and Epinephrine s.c.; 15 Weeks	15 Weeks	④⑥	Elevated Colonic Mucosal Damage Index (CMD1)	Cellular immune hyperfunction and humoral and non-specific immune dysfunction	Chaihu Shugan Powder	Yao et al. (2006)
Spleen Deficiency Syndrome		BALB/CMice	CT26 Cell s.c. with Senna decoction i.g.; 4 Weeks	4 Weeks				Jianpi Jiedu Decoction	Chen et al. (2018)
		BALB/CMice	Spleen Injection of CT26 Cell with Hemi-spleen and Decoction of raw Rhubarb i.g.; 10 Days	10 Days	⑥	Ulcerative adenocarcinoma with increased depth of tumor invasion		Jiawei Sijunzi Decoction	Qiao (2020)
Blood stasis Syndrome		BALB/CMice	Spleen Injection of LoVo Cells to Induce Hepatic Metastasis Model with Fludrocortisone IM and Adrenaline s.c.; 24 Days	24 Days	⑤		Increased blood viscosity	Stasis-Resolving and Toxin-Dispelling Formula	Jia and Huang (2007)
	with coagulated cold syndrome	BALB/CMice	C26 Cells s.c. Administered in Two Doses with a 1-Hour Interval, First at Room Temperature, Second with Cold Bath; 4 Weeks	4 Weeks			Elevated expression of MMP-2 and VEGF		Zhou and Guo (2012)
	with qi deficiency syndrome	BALB/CMice	C26 Cell s.c. with Reserpine IM; 42 Days	42 Days	⑤⑥		Increased blood rheology score	Sijunzi Decoction combined with Blood Stasis-Expelling Decoction	Qian et al. (2016)
	with heat-toxin syndrome	BALB/CMice	C26 Cell s.c. with LPS i.v.; 6 Weeks	6 Weeks	①⑤		Blood is in a highly viscous state	Blood Stasis-Expelling Decoction combined with Rhinoceros Horn and Rehmannia Decoction	Li et al. (2017)
Dampness-Heat Syndrome	with the syndrome of static blood and toxin	C57BL/6N Mice	AOM i.p. and DSS Free Drinking with High-Fat Diet and Damp-Heat Atmosphere; 28 Weeks	28 Weeks		Loss of epithelial polarity showing nest-like/cribriform patterns with central necrosis, increased nuclear-cytoplasmic ratio, hyperchromatic nuclei, prominent nucleoli, and increased mitotic activity	Related to the classification and activity of various cells in the tumor microenvironment	Xianlian Jiedu Decoction	Zhang et al. (2022a)
		C57BL/6Mice	AOM i.p. and DSS Free Drinking with Damp-Heat Atmosphere; 16 Weeks	16 Weeks	①③		Elevated levels of ALB, IL-17, and IL-10 in serum	Huang Qin Tang combined with Actinidia root	Huang et al. (2022)
		BALB/CMice	The of *In Situ* Transplantation CT26 Cell with High-Fat, High-Sugar Diet and white spirit i.g.; 3 Weeks	3 Weeks			Increased expression of Ki67 and MVD	Spleen-strengthening and Detoxification Prescription	Liu et al. (2015)

^a^ Syndrome evaluation indicators:① Heat pattern:Irritability, hyperactivity, elevated body temperature, increased water intake, reddish discoloration of ear margins, nail beds, and perioral regions, dry/hard or blood-streaked stools, and perianal erythema; ② Cold pattern:Aversion to cold, diarrhea, decreased body temperature, reduced water intake, pale or cyanotic lips/ear margins, pale nail beds; ③ Dampness pattern:Weight gain, greasy/dull fur, huddling behavior, reduced locomotor activity, sluggish responsiveness, turbid genital secretions, and loose/sticky stools; ④ Depression pattern:Decreased locomotor activity, reduced aggression, heightened startle response, hunched posture, and reduced food intake; ⑤ Blood stasis pattern: Purplish-red discoloration or ecchymosis on ear margins, nail beds, abdominal wall, gingiva, perianal/perioral regions, dry/brittle fur with alopecia; ⑥ Spleen deficiency pattern:Weight loss, reduced food consumption, lethargy, huddling behavior, sluggish responsiveness, abdominal distension, loose stools, and dry/yellowish fur.

Furthermore, the current repertoire of CRC models integrating disease and syndrome in TCM remains limited. Most of these models have yet to establish widely accepted and reproducible modeling methodologies, indicating that this field is still in its nascent exploratory phase. Future research should focus on optimizing the existing techniques and methods for constructing CRC disease-syndrome combination models. Efforts should be directed toward enhancing the diversity of syndrome-specific models and refining the evaluation criteria for these models. The ultimate goal is to develop highly reliable, stable, and reproducible CRC animal models that align with the principles of TCM syndrome differentiation and treatment, thereby advancing their applicability in both research and clinical practice.

## 4 Application of CRC mouse models

As a bridge to clinical application, CRC mouse models have been widely used in various aspects of CRC therapy (Kucherlapati, 2023). Different transplant mouse models have their unique characteristics, and likewise, each model has its drawbacks. Currently, the applications of CRC mouse models mainly involve the development of biomarkers, drug testing, and surgical modeling. For example, subcutaneous transplantation models have limited predictive value for human clinical response through large drug screening. In contrast, the orthotopic PDX models maintain the highest concordance of drug responses between patients and mouse models, supporting their use as an optimal screening platform for anticancer drug evaluation (Xie and Lin, 2020). Currently, the applications of CRC mouse models mainly involve the development of biomarkers, drug testing, and surgical modeling (Oliveira et al., 2020).

### 4.1 Investigation of the pathogenesis

Typically, the occurrence and development of human CRC can be simulated by CRC murine models, which offer researchers a platform to develop a comprehensive comprehension of the mechanisms of tumor formation. By constructing primary tumor models, such as chemically induced models and genetically engineered mouse models, researchers can explore how various carcinogenic factors affect the normal physiological processes of intestinal cells, ultimately leading to the formation of tumor. Undoubtedly, these models serve as a theoretical foundation for the development of effective prevention and treatment by revealing the multi-step, multi-factorial processes of tumorigenesis (Pothuraju et al., 2024).

Beyond these, CRC mouse models are also considered useful tools for studying gut microbiota in CRC development. In our previous studies, we also demonstrated that microecological imbalances can promote colorectal tumorigenesis (Sui et al., 2020b; Zhang et al., 2023). Thus, microecological imbalance is complementary and causally related to the development of colorectal cancer.

### 4.2 Development of biomarkers

CRC mouse models, especially the PDX model, can reveal novel biomarkers, thus providing reliable evidence for individualized treatment of CRC patients. In a recent study, amplification of the human epidermal growth factor receptor 2 (HER2) gene was shown to promote cetuximab resistance in a KRAS/NRAS/BRAF/PI3KCA wild-type PDX model with metastatic CRC and was found to predict responses to antiepidermal growth factor receptor (EGFR) and anti-HER2 antibodies (Gupta et al., 2024). Subsequently, these findings were further translated into successful clinical studies. In line with this, other researcher found that EGFR-inhibited surviving metastatic CRC cells exhibited reduced EGFR ligand expression, enhanced HER2/HER3 signaling pathway activity, and sustained activation of the phosphatidylinositol 3-kinase (PI3K) pathway in the PDX model (Venturini et al., 2024).

In summary, the discovery of novel tumor biomarkers with the help of CRC mouse models is essential in promoting translational research in clinical and basic sciences. Moreover, it can facilitate individualized treatment based on tumor molecular classification.

### 4.3 Drug testing

Transplant mouse models can be used in investigating the efficacy of controversial or novel antitumor agents against CRC. Using the CDX model, Liu et al. demonstrated that a novel andrographolide derivative AGS-30 could induce apoptosis in CRC cells by activating the ros-dependent JNK signaling pathway (Liu et al., 2022). Through the CDX model, our group found that the traditional Chinese medicine sophocarpine can enhance the inhibitory effect of oxaliplatin on metastatic CRC (Sui et al., 2016; Sui et al., 2014).

Another application of CRC mice models is the co-clinical trial, which is defined as a clinical trial conducted in parallel with a preclinical trial. Currently, co-clinical trials in CRC have already generated many hopeful outcomes. For example, in BRAF-mutated CRCs, PDX models were identified to faithfully replicate clinical results and permit further study of acquired resistance mechanisms (Liu et al., 2022). Therefore, the establishment of PDX models from clinical trial participants, followed by treatment with novel therapeutic agents, enables the identification of prognostic biomarkers and facilitates the investigation of underlying mechanisms of drug response.

## 5 Conclusion

The development and refinement of preclinical rodent models have been pivotal in dissecting the molecular complexity of CRC and accelerating therapeutic discovery. As highlighted in this review, each model system (from carcinogen-induced models and GEMMs to PDX platforms and metastatic/spontaneous models) offers unique advantages tailored to specific research goals. Conventional models such as GEMMs continue to illuminate the genetic and signaling cascades driving tumor initiation, while PDX models have emerged as indispensable tools for capturing interpatient heterogeneity and evaluating patient-specific therapeutic vulnerabilities. Meanwhile, composite models bridge the gap between reductionist approaches and clinical reality by integrating multifactorial tumor-host interactions.

Despite these advances, critical challenges persist. Most models fail to fully recapitulate the human tumor immune microenvironment, and species-specific disparities in drug metabolism may limit translational predictability. Furthermore, the high cost and technical demands of advanced models like PDX necessitate strategic prioritization of resources. Future efforts should focus on engineering immunocompetent PDX systems through humanized mouse strains, leveraging CRISPR-Cas9 to create multigenic spontaneous models, and combining longitudinal imaging with multi-omics profiling to capture dynamic tumor evolution.

Importantly, no single model can fully encapsulate the spectrum of CRC biology. A synergistic approach-matching experimental objectives to model strengths-will remain essential. For instance, GEMMs may guide mechanistic hypothesis generation, whereas PDX cohorts could validate candidate therapies in clinically relevant contexts. As the field moves toward precision oncology, the integration of these models with emerging technologies (e.g., organoid-based platforms, AI-driven drug screening) promises to unlock deeper insights into metastatic resistance and immune evasion. Collectively, continued innovation in preclinical modeling will not only refine our understanding of CRC pathogenesis but also catalyze the transition from empirical treatment paradigms to biomarker-driven, patient-tailored therapies.
